# Rapid Detection of Anti-SARS-CoV-2 Antibody Using a Selenium Nanoparticle-Based Lateral Flow Immunoassay

**DOI:** 10.1109/TNB.2021.3105662

**Published:** 2021-08-18

**Authors:** Chunxia Chen, Hangzhan Hu, Xiaoquan Li, Zhi Zheng, Zhizeng Wang, Xuance Wang, Peiming Zheng, Facai Cui, Gang Li, Yaohui Wang, Zhigang Liu, Yuanfang Ma

**Affiliations:** Joint National Laboratory for Antibody Drug Engineering, Clinical Laboratory of the First Affiliated Hospital, School of MedicineHenan University 12411 Kaifeng 475004 China; School of Basic Medical of SciencesHenan University 12411 Kaifeng 475004 China; Joint National Laboratory for Antibody Drug Engineering, Clinical Laboratory of the First Affiliated HospitalHenan University 12411 Kaifeng 475004 China; Department of Clinical LaboratoryHenan Provincial People’s HospitalHenan University 12411 Zhengzhou 450003 China

**Keywords:** SARS-CoV-2, COVID-19, selenium nanoparticle, rapid detection, antibody

## Abstract

Coronavirus disease 2019 is an infectious disease caused by the severe acute respiratory syndrome coronavirus 2 (SARS-CoV-2). SARS-CoV-2 is highly transmissible. Early and rapid testing is necessary to effectively prevent and control the outbreak. Detection of SARS-CoV-2 antibodies with lateral flow immunoassay can achieve this goal. In this study, SARS-CoV-2 nucleoprotein (NP) was expressed and purified. We used the selenium nanoparticle as the labeling probe coupled with the NP to prepare an antibody (IgM and IgG) detection kit. The detection limit, cross reaction, sensitivity and specificity of the kit is verified. Separate detection of IgM and IgG, such as in this assay, was performed in order to reduce mutual interference and improve the accuracy of the test results.The final purity of NP was 91.83%. Selenium nanoparticle and NP successfully combined with stable effect. The LOD of the kit was 20 ng/mL for anti-NP IgG and 60 ng/mL for anti-NP IgM, respectively. The kit does not cross reaction with RF. The sensitivity of the kit was 94.74% and the specificity was 96.23%. The assay kit does not require any special device for reading the results and the readout is a simple color change that can be evaluated with the naked eye. This kit is suitable for rapid and real-time detection of the SARS-CoV-2 antibody IgG and IgM.

## I. Introduction

Beginning in December 2019, several unexplained pneumonia cases occurred in Wuhan City, Hubei Province. Chinese researchers identified the cause of the disease as a new type of coronavirus, namely severe acute respiratory syndrome coronavirus 2 (SARS-CoV-2) [1]. The virus belongs to the $\beta $-genus of the coronavirus family, which also includes the SARS coronavirus responsible for the outbreak in 2003 and the MERS coronavirus responsible for the outbreak in 2012. The virus is listed by the World Health Organization as the seventh coronavirus known to infect humans [2]. SARS-CoV-2 contains spike protein, envelope protein, membrane protein, and nucleoprotein. Of these proteins, nucleoprotein is the most highly conserved [3]. The incubation period after infection is 0–24 days, and all symptomatic infections involve pneumonia. Common symptoms are fever, cough, and myalgia or fatigue [4], and less common symptoms include expectoration, headache, hemoptysis, diarrhea, and dyspnea. The symptoms are similar to the common cold; however, if the patient is not diagnosed and treated in time, the disease can be fatal [1], [2]. SARS-CoV-2 is transmitted through droplets in the air, and current data do not exclude the possibility of fecal-oral transmission [5]. There is no age preference for viral infection [6]. Infections can be in family clusters, and there are also asymptomatic infections [7]. At present, more than 210 countries and regions have reported confirmed cases of the coronavirus disease 2019 (COVID-19) caused by SARS-CoV-2. SARS-CoV-2 has a long incubation period, a wide range of transmission channels, and screening is required for a large population, all of which increases the difficulty in creating an optimal detection assay. On August 19, 2020, the General Office of the National Health Commission and the Office of the State Administration of Traditional Chinese Medicine jointly issued the Diagnosis and Treatment Program for COVID-19 (trial version 7) [8] Gene sequencing and antibody (IgM/IgG) test results are recommended to be used as the basis for the diagnosis. In addition, it is widely accepted that IgM provides the first line of defense during viral infections, before the generation of adaptive, high affinity IgG responses [9]. When a pandemic outbreak occurs, portable, fast, and real-time screening methods are needed to help prevent and control the pandemic; a lateral flow immunoassay (LFIA) can meet these requirements.

The LFIA is a fast, simple, accurate, stable, and portable diagnostic tool [10], [11]. LFIAs utilize various types of labeling probes that either provide quantitative detection or qualitative detection [12]. Quantitative detection labeling probes include fluorescent microspheres [13], upconversion luminescence [14], quantum dots [15], [16], and magnetic particles [17]; qualitative detection labeling probes include colloidal gold [18], selenium nanoparticle [19], and nanocarbon [12]. Quantitative detection is the trend for these types of assays, but the drawback is that these methods do not provide a readout that is visible with the naked eye, and additional equipment is required to interpret the results. This practical limitation prevents the use of most quantitative lateral chromatography-based assays in SARS-CoV-2 screening. Currently, home self-tests are still mostly qualitative tests. Colloidal gold is the most widely used qualitative labeling probe [18]. Selenium nanoparticles are another labeling probe used in lateral chromatography experiments. The nanoparticles have a surface plasmon effect, a small-size effect, and are used to label proteins or nucleic acids. Positive test results appear as orange lines visible to the naked eye because selenium nanoparticles are orange colored [19]. Selenium nanoparticles are prepared at room temperature, easily bind to proteins, and are not sensitive to electrolytes. They also have higher levels of sensitivity, and are more economical to prepare than other probe types [20]. The current study is based on our reported article on how to prepare selenium nanoparticles [19]. Here, we apply this technique to the development of a rapid detection kit for anti-SARS-CoV-2 IgM and IgG based on selenium nanoparticle probes. In the test, we separately detect IgM and IgG in order to reduce mutual detection interference and improve the accuracy of test results.

## II. Materials and Methods

### A. Materials and Apparatus

Polyethylene glycol (PEG) was purchased from the YuanYe Biological Technology Co., Ltd (Shanghai, China). Ascorbic acid (Vc) and selenous acid (H_2_SeO_3_) were obtained from Xiya Reagent (Shandong, China). Bovine serum albumin (BSA) was purchased from Sigma. The anti-NP IgM and anti-NP IgG antibodies (Abs) were acquired from the Beijing Biodragon Immunotechnologies Co. Ltd (Beijing, China). Deionized water (Millipore Milli-Q Grade) with a resistivity of 18.2 $\text{M}\Omega \cdot $cm was used throughout this work. The dispenser and sprayer XYZ 3010 and the guillotine cutter were provided by the Shanghai JieNing Biological Technology Co., Ltd (Shanghai, China). An electric blast drying oven was provided by Shanghai YiHeng Scientific Instruments Ltd (Shanghai, China).

### B. Expression and Purification of SARS-CoV-2 Nucleoprotein

The pET28a(+)-nCoV-nucleoprotein-histidine tag plasmid was constructed, and the SARS-CoV-2-N-His recombinant protein was produced using E. coli BL21. The plasmid was transformed into E. coli BL21, and a single clone was inoculated into 5 mL of 2YT medium and cultured at 37°C and 220 rpm for 14 h. Subsequently, 2 mL of the bacterial solution was added into 1 L of 2YT medium and cultured at 37°C and 150 rpm for 2.5 h. The bacterial broth was incubated with 0.1 mM isopropyl $\beta $-D-1-thiogalactopyranoside (IPTG) at 30°C and 150 rpm for 6 h. The bacterial solution was centrifuged, the culture medium discarded, and the bacteria fully resuspended in 50 mL of phosphate-buffered saline (PBS). This solution was sonicated with a cell ultrasonic disruptor, according to the following protocol: 250 W ultrasound for 10 s, pause for 10 s, for 30 cycles, at which time the bacterial solution became transparent. After sonication, the bacterial solution was centrifuged at 15,000 rpm for 20 min, and the supernatant was filtered through a $0.22~\mu \text{m}$ filter membrane, followed by affinity purification using a His Trap FF crude 5 mL nickel column. The purity of nucleoprotein was analyzed by sodium dodecyl sulfate polyacrylamide gel electrophoresis (SDS-PAGE) and quantified using high performance liquid chromatography (HPLC). The concentration of nucleoprotein was measured by BCA protein assay kit.

### C. Preparation of Selenium Nanoparticles and Recombinant Nucleoprotein Complex

The selenium nanoparticle solution (SeNps) was prepared using ascorbic acid to reduce selenite. The selenium nanoparticle preparation was used to label recombinant nucleoproteins at different pH levels, and the pH of SeNps was adjusted with K_2_CO_3_. Then, recombinant nucleoprotein was added in drops to 100 mL SeNps with gentle stirring at room temperature. The mixture was then softly agitated for 30 min to allow conjugation of recombinant nucleoproteins to selenium nanoparticle surfaces by physical adsorption. Subsequently, 1 g bovine serum albumin (BSA) was added to the mixture for 30 min to block the non-coated selenium nanoparticle surfaces, and then the mixture was centrifuged at 10,000 rpm for 10 min. Selenium nanoparticle-conjugated recombinant nucleoprotein in a soft-pellet form was collected. Finally, the labeled proteins were resuspended in a working solution (pH 7.4 10 mM PBS containing 0.05% Tween 20, 1% BSA, 5% sucrose, and 5% trehalose) and then stored at 4°C before use. The optimal pH was selected according to color development. At the selected optimal pH, 1.5 mg/mL, 2 mg/mL, and 4 mg/mL were selected as antibody coating concentration at the test line. $5~\mu \text{g}$/mL, $10~\mu \text{g}$/mL, and $20~\mu \text{g}$/mL were the alternative conjugating concentrations of the recombinant nucleoproteins. Binding efficiency is measured by transmission electron microscope (TEM) and dynamic light scattering (DLS). 1 mg/mL was used as the coating concentration of the anti-His antibody at the control line to prepare the kit, as the recombinant nucleoprotein of SARS-CoV-2 has His tag. The criteria for determining the optimal conditions were the number of false positive results and the degree of color development.

**Fig. 1.**
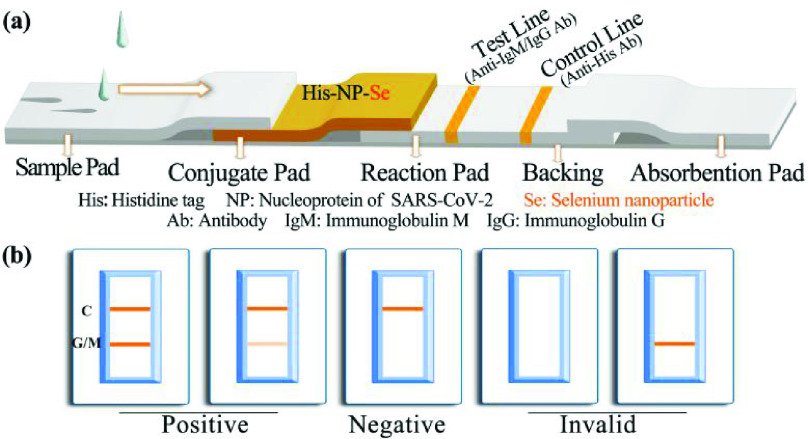
Diagram and components of the SARS-CoV-2 antibody immunoassay test strip (a) and visual assessment guidelines for interpreting the test strip results (b). Abbreviation: SARS-CoV-2, severe acute respiratory syndrome coronavirus 2; C: Control line; G: Test line for IgG detection; M: Test line for IgM detection.

The internal structure of the kit is shown in Figure 1a and includes a backing, a sample pad, a conjugate pad, a reaction pad, and an absorbent pad. The liquid matrix added to the sample pad is chromatographed to the conjugate pad to re-dissolve the selenium-labeled protein complex fixed to the conjugate pad, and, due to the capillary action, the selenium-labeled recombinant nucleoprotein is chromatographed with the liquid matrix toward the absorbent pad in the nitrocellulose membrane. The process for IgM detection is provided as an example in Figure 1b. When the sample contains anti-nucleoprotein of SARS-CoV-2 IgM, the IgM conjugates to the selenium nanoparticle-labeled recombinant nucleoprotein. When this protein complex conjugate is chromatographed to the position of the test line, the IgM will bind to the anti-human IgM antibody, resulting in the accumulation of color at the position of the test line. When the excess complex conjugate is chromatographed to the control line area, the anti-His antibody coated in this region captures the selenium nanoparticle-labeled His-tag nucleoprotein, resulting in color accumulation. Therefore, when both the control line and the test lines develop color, it is determined that the sample contains anti-nucleoprotein IgM, and the test result is considered positive. When a negative sample is applied to the test, there is no immune binding reaction in the detection area. Thus, no color will be accumulated at the test line, but the control line will develop color normally. Therefore, when the control line develops color and the test line does not develop color, it is determined that the sample does not contain anti-nucleoprotein IgM, and the test result is considered negative. If the control line does not develop color, the condition of color development at the test line is an invalid result. Interpretation of IgG test results works in the same way as for IgM. For the anti-SARS-CoV-2 IgG test, the anti-human IgG antibody was coated at the position of the test strip, and other the conditions remained unchanged.

### D. Preparation of the SARS-CoV-2 Antibody Detection Kit

The anti-human IgM antibody or anti-human IgG antibody was sprayed on the test area as M line or G line by dispenser and sprayer (XYZ 3010), and the anti-His antibody was coated on the control line. After the coating was completed, the reaction pad was dried at 37°C for 4 h. The sample pad was immersed in the sample pad treatment solution (10 mM PBS, pH 7.4, containing 0.05% Tween 20, and 5% serum) for 30 min and dried at 37°C for 4 h. The conjugate pad contained immobilized selenium nanoparticle recombinant nucleoprotein complex. The test strips were prepared prior to performing the assay, assembled, cut, and ready for use.

### E. Determination of the Detection Limit and Cross Reaction of the Anti-SARS-CoV-2 Detection Kit

We got commercially calibrated antibodies (Beijing Biodragon Immunology Technology Co., Ltd., BF03088, BF03089) diluted in negative serum as standards. Samples containing different concentration of anti-NP IgG (0–1000 ng/mL) anti-NP IgM (0–1000 ng/mL) in negative serum were applied on the sample pad for the limit of detection (LOD) assay in triplicate. The results could be determined by the naked eye within 10 min. At the same time, the test results were analysed by ImageJ and GraphPad Prism 8 to assist in determining the LOD. The detection of human IgG and IgM is easily affected by rheumatoid factors (RF), so it was necessary to test whether the kit has cross reaction with RF. Different concentration of RF (0–50U) were detected by this lateral flow kit.

### F. Clinical Sample Validation

Fetal bovine serum (FBS) was chosen as the negative sample for the selection of kit conditions in the early stage. The serum of two healthy persons was used to verify the above conditions. SARS-CoV-2 positive specimen and negative specimen were used to validate the test strips. Among them, 19 cases were diagnosed as positive in Henan Provincial People’s Hospital, the COVID-19 designated hospital in Henan Province; 41 cases were confirmed negatives by clinical detection from the First Affiliated Hospital of Henan University, 12 normal persons who had no cold or fever symptoms within 2 weeks were also recruited into the study. For each kit, $16~\mu \text{L}$ of plasma and $64~\mu \text{L}$ of sample processing solution were added. The sample processing solution was 10 mM PBS containing 0.05% Tween-20 and 1% BSA, pH 7.4, and the results were observed after 10 min. The observation results are shown in Figure 1. When both the test line and the control line develop color, the result is positive; when only the control line develops the color, the result is negative; when the control line does not develop a color, the result is invalid. We confirm that the detection results of COVID-19 patients in this study have not been reported in any other submission by us or anyone else.

The sensitivity, specificity, positive predictive value, and negative predictive value of the kit were calculated according to the following formulas [21]:

\begin{align*} \text {Sensitivity}=&\Biggl[\text {True Positive}/\Biggl(\text{True Positive} \\&+\, \text {False Negative}\Biggr)\Biggr] \times 100{\%}\\ \text {Specificity}=&\Biggl[\text {True negative} / \Biggl(\text {True Negative} \\&+\, \text {False Positive}\Biggr)\Biggr] \times 100{\%}\\ \text {Positive predictive value}=&\Biggl[\text {True Positive} / \Biggl(\text {True Positive} \\&+\, \text {False Positive}\Biggr)\Biggr] \times 100{\%}\\ \text {Negative predictive value}=&\Biggl[\text {True Negative} / \Biggl(\text {False Negative} \\&+\, \text {True Negative}\Biggr)] \times 100{\%}\end{align*}

## III. Results

### A. Expression and Purification of SARS-CoV-2 Nucleoprotein

**Fig. 2.**
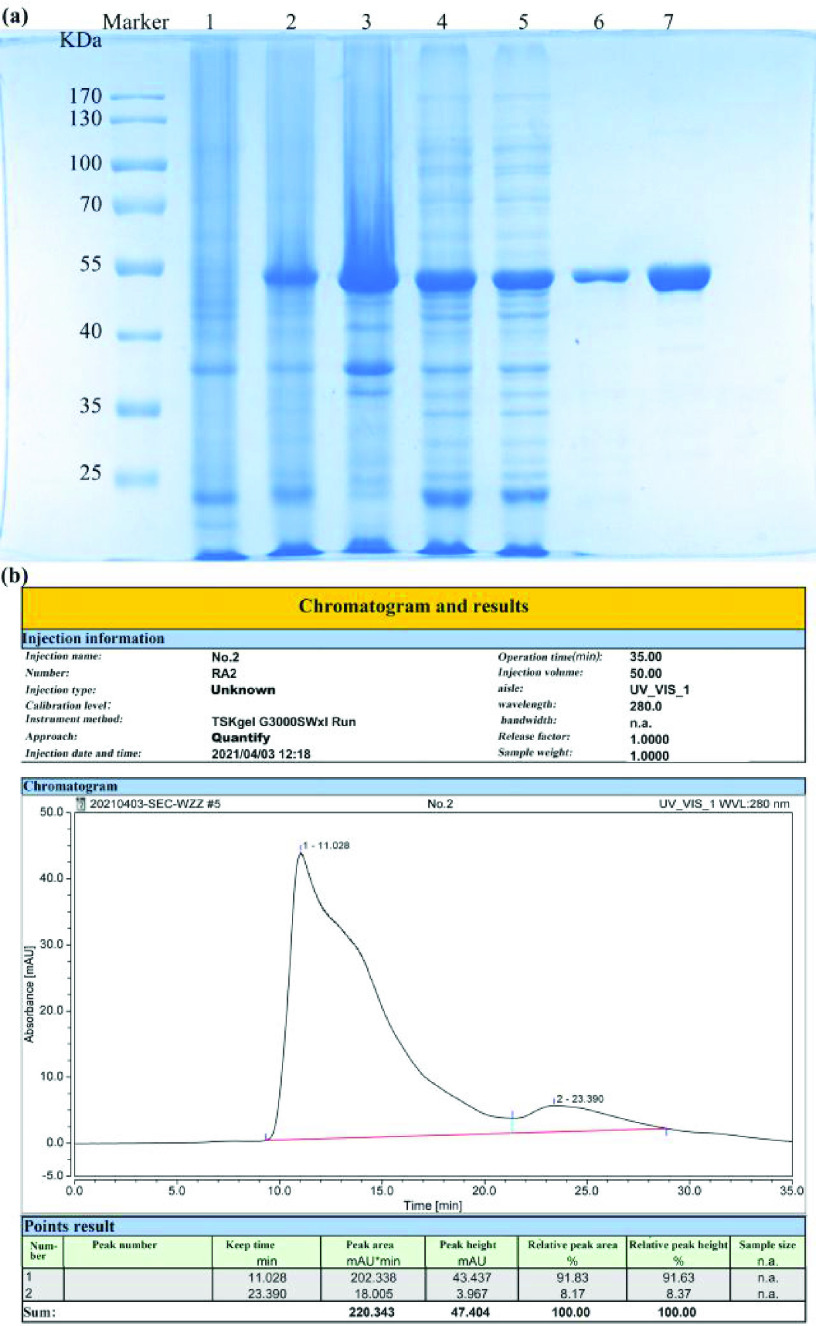
SARS-CoV-2 recombinant nucleoprotein purification results. (a) SDS-PAGE, (b) Purity verification byHPLC, Abbreviation: 1) Bacterial culture before induction. 2) Bacterial culture after induction. 3) Precipitate from centrifuged bacterial culture after induction. 4) Supernatant from centrifuged bacterial culture after induction. 5) Flow-through of nickel column supernatant. 6) Eluate of the target protein—nucleoprotein conjugate. 7) The nucleoprotein by ion exchange. SARS-CoV-2, severe acute respiratory syndrome coronavirus.

As shown in Figure 2, after IPTG induction of protein production in E. coli, nucleoproteins were expressed and detected at approximately 50 kD (Figure 2a). The target protein was present in the supernatant and the precipitate after sonication of the bacterial solution. The target protein was purified by affinity chromatography on a nickel column, and the final purity was 91.83% (Figure 2b) tested by HPLC. As determined by the BCA kit, the protein concentration was 1.22 mg/mL, which was sufficient yield for the preparation of the kit.

### B. Preparation and Validation of Selenium Nanoparticle Anti-SARS-CoV-2 Detection Kit

**Fig. 3.**
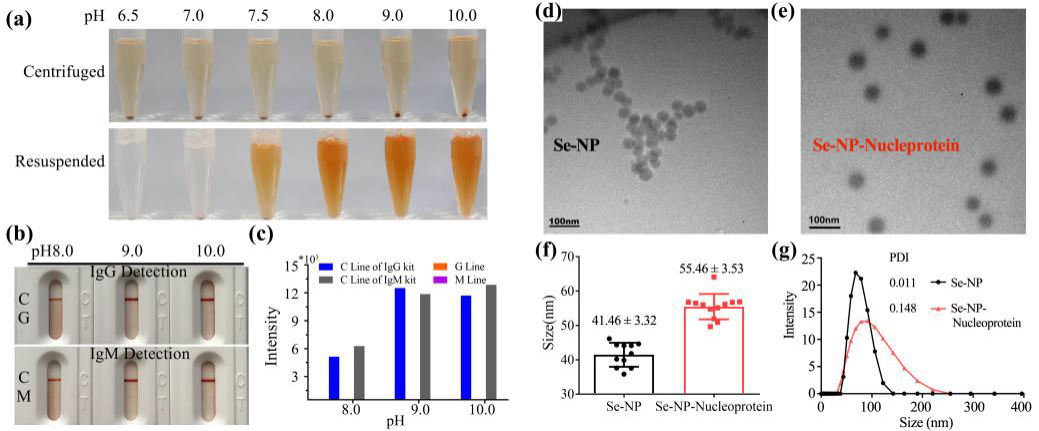
pH value determination for selenium nanoparticle-conjugated recombinant nucleoprotein. Photographs of centrifuge, resuspending (a) the test strip naked results (b), auxiliary judgment result (c), morphology of Se-NP (d), Se-NP-nucleoprotein (e), size analysis (f) and combined effect (g) are shown. C: Control line; G: Test line for IgG detection; M: Test line for IgM detection; PDI: Polymer dispersity index; Se-NP: Selenium nanoparticle.

The conjugating concentration is the amount of nucleoprotein added to the selenium nanoparticle solution per unit volume. The coating concentration is the concentration of antibody sprayed on the test line. Nucleoprotein was labeled with selenium nanoparticles as follows. The normal conjugating concentration was $10~\mu \text{g}$/mL, and the coating concentration was 2 mg/mL. The selected test pH levels were 6.5, 7.0, 7.5, 8.0, 9.0, and 10.0. The protein was centrifuged and resuspended after labeling. At pH 6.5, 7.0, and 7.5, the protein stuck to the test tube bottom and was difficult to resuspend, especially at pH 6.5 and 7.0 (Figure 3a). Therefore, pH 8.0, 9.0, and 10.0 were selected for subsequent experiments. During serum testing, no false positives were identified at pH 8.0, 9.0, or 10.0, irrespective of whether the IgG detection kit or IgM detection kit was used (Figure 3b). However, the color rendering effect and intensity of pH 8.0 was slightly worse than at pH 9.0 or 10.0 (Figure 3c). Based on these results, as well as the predicted isoelectric point of nucleoprotein, pH 10.0 was finally selected as the conjugating pH for selenium nanoparticle-labeled recombinant nucleoprotein. The particle size increased after labeling from 41.46 ±3.32 nm to 55.46 ± 3.53 nm by TEM (Figure 3d, 3e), indicating that the selenium nanoparticle and nucleoprotein labeling is successful, meanwhile, the conjugated complex was still evenly dispersed after marking. The polymer dispersity index value of selenium nanoparticle and conjugates is less than 0.2 by DLS, which means the conjugate is stable (Figure 3g).

**Fig. 4.**
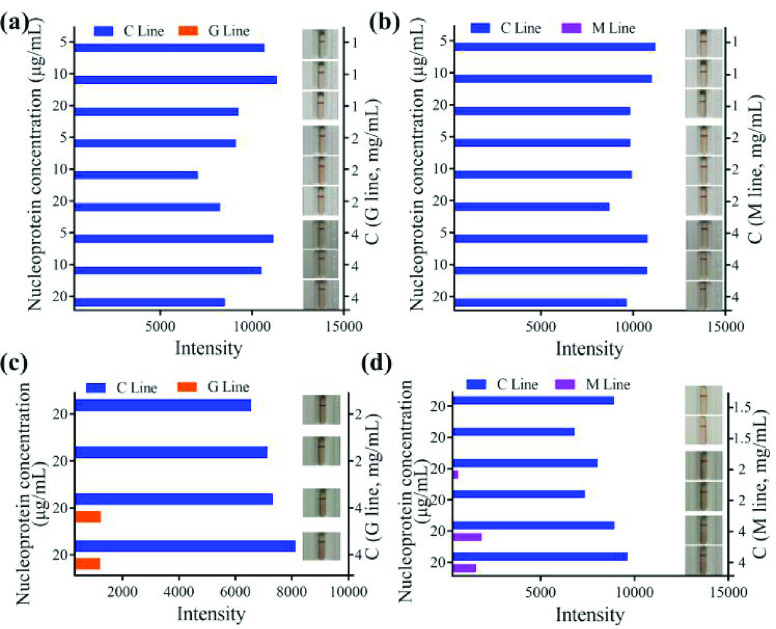
Selection of conjugating and coating conditions. Detection result for IgG (a) and IgM (b) with fetal bovine serum; detection result for IgG (c) and IgM (d) with two health human serum; C line: Control line; G line: Test line for IgG detection; M line: Test line for IgM detection; C: Concentration.

Using pH 10.0 as the conjugating condition, various conjugating concentrations and coating concentrations were tested. When the conjugating concentrations were $5~\mu \text{g}$/mL, 10 $\mu \text{g}$/mL, and $20~\mu \text{g}$/mL and coating concentrations were 1 mg/mL, 2 mg/mL, and 4 mg/mL, no false positive results occurred (Figure 4a, 4b; Figure S1a, 1b).

The SARS-CoV-2 antibody screening kit requires high sensitivity. A clinical sample validation study was therefore performed, using a conjugating concentration of $20~\mu \text{g}$/mL and a coating concentration of 2 and 4 mg/mL to screen for false positive results. Two negative samples were selected for testing. It was found that when the coating concentration was 4 mg/mL, both the IgG and IgM tests showed false positives (Figure 4c, 4d, Figure S1c, 1d). When the coating concentration was 2 mg/mL, the IgG test showed no false positive results (Figure 4c, Figure S1c), but a weak false positive result occurred in the IgM test (Figure 4d, Figure S1d). When the coating concentration was 1.5 mg/mL, there was no false positive result in the IgM test (Figure 4d, Figure S1d). Therefore, 1.5 mg/mL and 2 mg/mL coating concentrations were selected for anti-IgM and IgG antibodies, respectively. A final conjugating concentration of $20~\mu \text{g}$/mL nucleoprotein was selected for use in the final product, as well as 1.5 mg/mL as the anti-IgM antibody coating concentration, 2 mg/mL as the anti-IgG antibody coating concentration, and 1 mg/mL as the anti-His antibody coating concentration.

### C. Limit of Detection and Cross Reaction of Determination

**Fig. 5.**
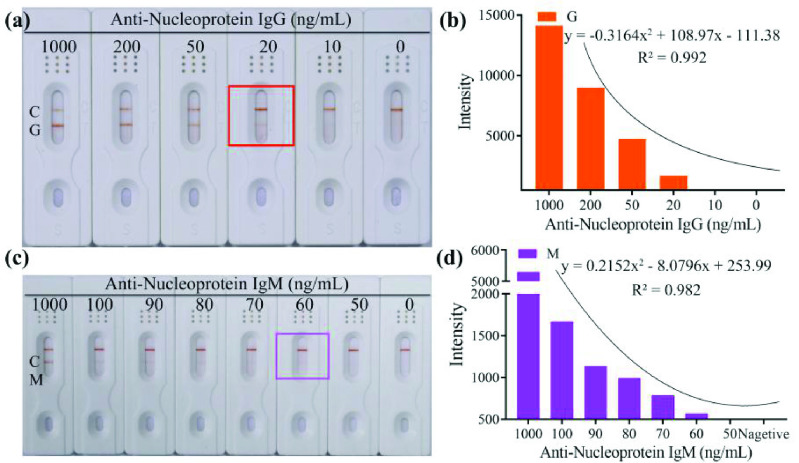
Limit of detection of the kit for IgG and IgM detection. Result of detection for IgG (a) and IgM (c) by naked eyes; Analysis result of detection for IgG (b) and IgM (d) by GraphPad Prism 8. C: Control line; G: Test line for IgG detection; M: Test line for IgM detection.

**Fig. 6.**
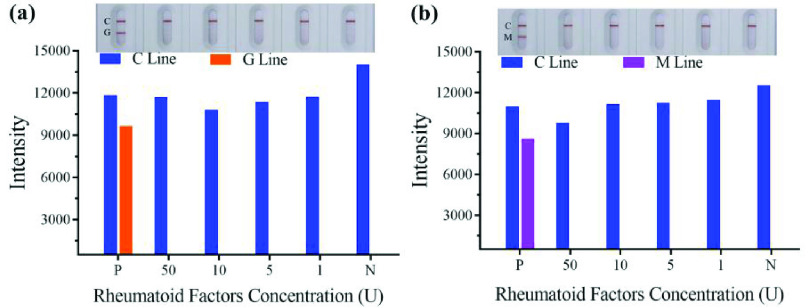
Cross reaction results of anti-SARS-CoV-2 IgG (a) and IgM (b) for rheumatoid factor. SARS-CoV-2, severe acute respiratory syndrome coronavirus 2. P: Positive; N: Negative; C: Control line; G: Test line for IgG detection; M: Test line for IgM detection.

Different concentrations of anti-nucleoprotein IgG (1000, 200, 50, 20, 10 and 0 ng/mL) anti-nucleoprotein IgM (1000, 500, 200, 100, 90, 80, 70, 60, 50 and 0 ng/mL) in negative serum were tested by the corresponding kit in triplicate. The analysis process was completed in less than 10 min, and the LOD of anti-nucleoprotein IgG was 20 ng/mL (Figure 5a, 5b) with fitting curve $\text{y}=-0.3164\times 2 +108.97\text{x}$ – 111.38 (R2=0.992), the LOD of anti-nucleoprotein IgM was 60 ng/mL (Figure 5c, 5d, Figure S2) with fitting curve y $= 0.2152\times 2$ – 8.0796x + 253.99 (R^2^ =0.982), respectively. The intensity of orange color was proportional to the anti-nucleoprotein IgG and anti-nucleoprotein IgM concentration in the range of 20–1000 ng/mL (Figure 5a) and 100–1000 ng/mL (Figure 5c). The kit does not cross-react with RF of not higher than 50U, when the positive control and different concentrations of RF adding the sample pad (Figure 6a, 6b).

### D. Clinical Specimen Validation

**Fig. 7.**
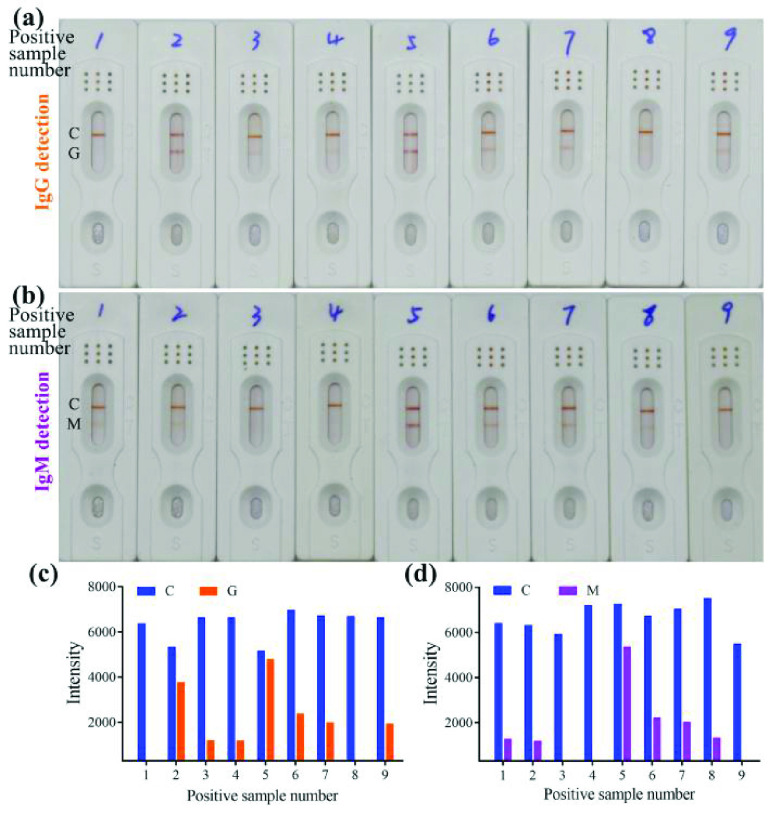
Clinical sample detection results of anti-SARS-CoV-2 IgG (a) and IgM (b) by naked eyes; Analysis result of detection for IgG (c) and IgM (d) by GraphPad Prism 8. SARS-CoV-2, severe acute respiratory syndrome coronavirus 2. C: Control line; G: Test line for IgG detection; M: Test line for IgM detection.

A total of 53 samples were collected from the hospital, school and clinically diagnosed as negative for SARS-CoV-2. 39 patients and 12 normal persons were found to be IgG or IgM negative by testing and 2 were positive, including one IgG-positive sample and one IgM-positive sample (Figure S5-8, Table S4-6). In addition, 18 cases were found to be positive using our kit (Figure 7, Figure S3, 4 Table S2, 3.) in the 19 samples that tested positive by nucleic acid testing (Table S1). Of the positive results (Table I), twelve were positive (66.7%) for IgG detection, eleven (61.1%) were positive for IgM detection, and five cases (27.8%) were double positive. From these data, the sensitivity of the kit is determined to be 94.74%, the specificity is considered 96.23%, the positive predictive value is 90%, and the negative predictive value is 98.08%.

**TABLE I:** Distribution of Detection Results

		Confirmed Diagnosis		
		COVID-19	Non-COVID-19	Total
Developed LFIA	Positive(G/M/Double)	18(12/11/5)	2(0/1/1)	20
	Negative	1	51	52
Total		19	53	72
Sensitivity $=18/19\times100$% = 94.74%				
Specificity $=51/53\times100$% = 96.23%				
Positive predictive value $=18/20\times100$% = 90%				
Negative predictive value $=51/52\times100$% = 98.08%				

COVID-19, coronavirus disease 2019; LFIA: lateral flow immunoassay; G: immunoglobulin G; M: immunoglobulin M.

## IV. Discussion

The structural proteins of SARS-CoV-2 include spike protein, envelope protein, membrane protein, and nucleoprotein. Spike protein infects host cells by binding to the human ACE2 receptor. This spike protein shows great variability, but the receptor is suitable for drug development; however, the sample detection accuracy is likely to decrease as the SARS-CoV-2 mutates. Nucleoprotein is the most highly conserved and strong immunogenic of these structural proteins, so the sample detection rate can be improved through use of nucleoprotein [22]. Selenium nanoparticle and colloidal gold are both qualitative nano-labeled probes. Colloidal gold conjugated with protein based on hydrogen bonding, hydrophobic interactions, van der Waals forces, etc. serves as evidence for the existence of the electric double layer [9]. Therefore, selenium nanoparticle may also bind to nucleoprotein through these forces.

Antibodies will not be produced until about 10 days after infection, so the detection results by this kit of samples within 10 days of infection may be negative. Anti-spike protein antibody and anti-nucleoprotein antibody could be produced and secreted in the body after infected by SARS-CoV-2; and the nucleoprotein was selected to prepare the detection kit for antibody of SARS-CoV-2, therefore, this kit may not be able to detect samples that are antibody positive for spike protein.

When the effect of coating concentration was tested, FBS was selected to ensure the stability of the detection matrix. The serum component of FBS is less than that in human serum component resulting in less interfering factors. The coating concentration of the test strip 2 mg/mL anti-human IgG antibodies and 4 mg/mL anti-human IgM antibodies did not have false positives. However, when human serum matrix was tested, it was found that the anti-human IgG antibodies coated with 4 mg/mL showed false positives. In addition, coating with 2 mg/mL and 4 mg/mL anti-human IgM antibodies both showed false positive results. Therefore, 1.5 mg/mL anti-human IgM antibody was used for coating in test line.

The detectable antibodies of SARS-CoV-2 include IgG and IgM, which can be completed using two kits separately or using one kit which including IgG and IgM test line. The detection speed and method of interpreting the kit results are the same. In the separate detection strategy, there is no mutual interference between the target IgM and IgG, and the accuracy is higher compared with the combined detection strategy. A combined test can reduce the sample volume but the process requirements are higher than those of using two separate tests. In order to quickly develop a SARS-CoV-2 antibody detection kit, we here selected the separate test method, and we planned to develop a test kit for combined detection. Many antibody detection studies for SARS-CoV-2 based on colloidal gold were reported, and the results were shown in Table II. The research results from Liu’s team have more application value than others according the sensitivity, specificity and sample size [9], [23]–[25]. But none of these studies provide data on detection limits for IgM or IgG. Anti-nucleoprotein antibodies are secreted after vaccination with attenuated vaccines [26], and detected whether antibodies are produced by this study kit, and providing guidance for vaccination programs. The response rate is 91.18% (31/34) for anti-nucleoprotein IgG or IgM (Figure S9) after the second vaccination.

**TABLE II:** Performance Comparison of Different Detection Methods

Nanoparticle	Time (min)	Sensitivity (%)	Specificity (%)	Limit of Detection LOD(ng $\cdot $ml^−1^)		Ref.
				IgG (ng/mL)	IgM (ng/mL)	
Gold	15	100	93.3	—	—	24
Gold	15	88.66	90.63	—	—	25
Gold	15–20	69.1	100	—	—	26
Gold	15	95.85	97.47	—	—	27
Selenium	10	94.74	96.23	20	60	This work

In this study, only preliminary testing of clinical samples was performed. A total of 19 positive samples were tested, and 18 cases were consistent with nucleic acid results. Of the 19 patients, 9 were male and 11 were female. They were all severe and critical condition patients. One patient was 20–30 years old, three patients were 40–50 years old, four patients were 50–60 years old, and 11 patients were over 60 years old. The older patients might have compromised immunity, making them prone to be infected. Seven patients had diabetes, five patients had coronary heart diseases, and four patients had high blood pressure. Four of them had both diabetes and coronary heart disease, suggesting that patients with these diseases may be more susceptible to COVID-19. The patient who had the negative result had chronic bronchitis. Whether chronic bronchitis affects the detection of SARS-CoV-2 requires further research. To determine the true effectiveness of the kit, it is still necessary to expand the test sample size to validate the overall sensitivity and specificity of the kit. Moreover, patients having different stages of COVID-19 and a wide range of ages need to be tested to determine whether the detection results of the kit are affected by those factors.

## V. Conclusion

We have developed a new assay for the rapid detection of SARS-CoV-2 antibody (IgM + IgG) using a selenium nanoparticle based method. The LOD was 20 ng/mL for anti-NP IgG and 100 ng/mL for anti-NP IgM, respectively. The sensitivity of the kit is 94.74% and the specificity is 96.23%. The assay is portable and fast. The results can be obtained within 10 min. The assay kit does not require any special device for reading the results and the readout is a simple color change that can be evaluated with the naked eye. This kit is suitable for rapid and real-time detection of the SARS-CoV-2 antibody.

## Supplementary Materials

Supplementary materials
