# Supplementary material for: Rapid Detection of Anti-SARS-CoV-2 Antibody Using a Selenium Nanoparticle-Based Lateral Flow Immunoassay
Source: IEEE Trans Nanobioscience. 2021 Aug 18;21(1):37–43. doi: 10.1109/TNB.2021.3105662 (PMC8905607; doi:10.1109/TNB.2021.3105662)
Supplement: Supplementary materials [file supp1-3105662.pdf]

Supplemental materials

Rapid detection of anti-SARS-CoV-2 IgM and IgG using a selenium nanoparticle-based lateral flow immunoassay

Chunxia Chen†, Hangzhan Hu†, Xiaoquan Li†, Zhizeng Wang*, Zhi Zheng, Xuance Wang, Peiming Zheng, Facai Cui, Gang Li, Yaohui Wang, Zhigang Liu* and Yuanfang Ma*.

Chunxia Chen, Hangzhan Hu, Zhizeng Wang, Zhi Zheng, Xuance Wang, Yaohui Wang, and Yuanfang Ma are with Joint National Laboratory for Antibody Drug Engineering, Clinical Laboratory of the First Affiliated Hospital, School of Medicine, Henan University; School of Basic Medical of Sciences, Henan University, Kaifeng, 475004, China(email: ccxchem@163.com, hzz3356@163.com, wzhzeng@126.com, zhengzhi0119@126.com, wangxuance@126.com, wangyaohui2001@sina.com, and mayf@henu.edu.cn).

Xiaoquan Li and Zhigang Liu are with Joint National Laboratory for Antibody Drug Engineering, Clinical Laboratory of the First Affiliated Hospital, Henan University Kaifeng, 475004, China(email: 706534930@qq.com and 13592144067@126.com).

Peiming Zheng, Facai Cui, Gang Li are with Department of Clinical Laboratory of Henan Provincial People' Hospital of Henan University, Zhengzhou, 450003, China(zpm8266@163.com, cuifacai1@163.com and ligang65587359@163.com).

*Correspondence author: Zhizeng Wang, Zhigang Liu and Yuanfang Ma.

† These authors contributed equally.

Contents

Number	Name	Page
1	Selection of conjugating and coating conditions for anti-SARS-CoV-2 detection	3
2	High concentration of anti-nucleoprotein IgM detection by the IgM kit	4
3	Basic information of clinical samples for figure 7 and Table 1	5-21
4	Detection result for serum after the second vaccine injection by this kit	22

1 Selection of conjugating and coating conditions for anti-SARS-CoV-2 detection for figure 4

Different concentration of anti-human IgG and anti-human IgM is coated on the test line as G line and M line, respectively. Different concentrations of nucleoprotein conjugated with selenium nanoparticle to prepare conjugate pads. The effect (Figure 1) is verified by two negative serums. Figure S1a is coating condition selection of IgG detection for Figure 4a in manuscript; figure S1b is coating condition selection of IgM detection for Figure 4b in manuscript; figure 1c is conjugating condition selection of IgG detection for Figure 4c in manuscript; figure S1dis conjugating condition selection of IgG detection for Figure 4d in manuscript.

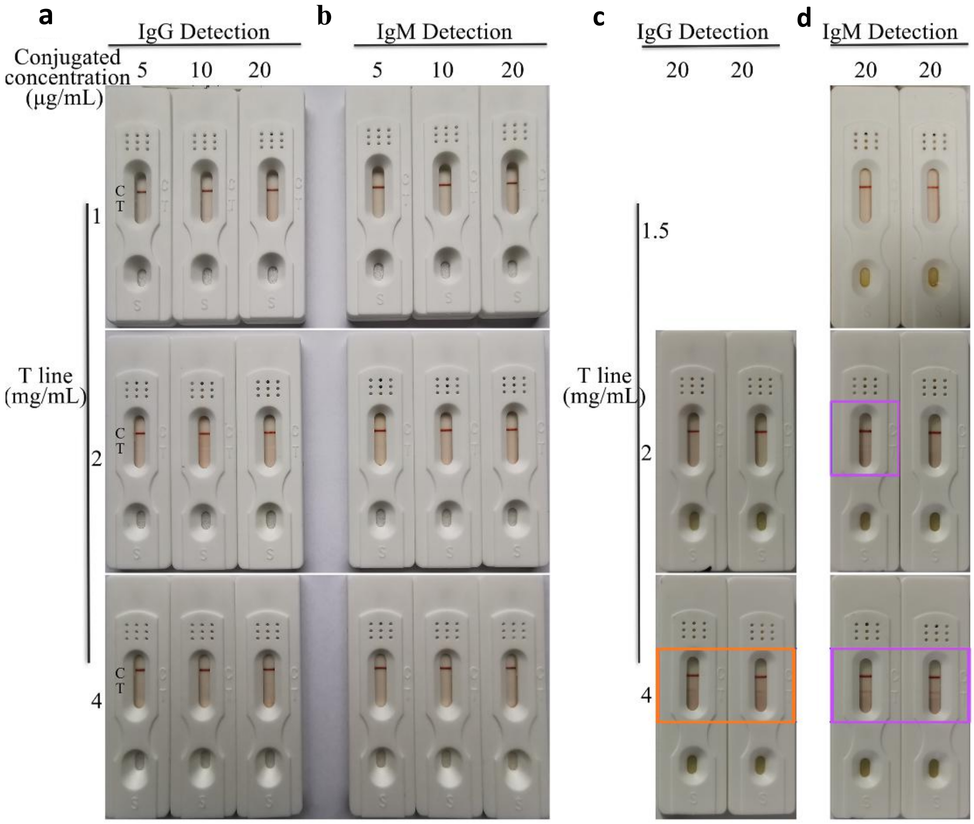

Figure S1 Selection of conjugating and coating conditions for anti-SARS-CoV-2 detection. SARS-CoV-2, severe acute respiratory syndrome coronavirus 2. Orange box: IgG false positive detection result; Purple box: IgM false positive detection result. C: Control line; T: Test line.

2 High concentration of anti-nucleoprotein IgM detection by the IgM kit High concentration (1000, 500, 200, 100, 50, 0 ng/mL) of anti-nucleoprotein of SARS-CoV-2 IgM was detected, and the result was shown in figure S2, the limit of detection (LOD) was 100 ng/mL by naked eye (figure S2a) and GraphPad Prism 8 (figure S2b); we added a set of detection (100, 90, 80, 70, 60, 50, 0 ng/mL of anti-nucleoprotein of SARS-CoV-2 IgM) as the 50 ng/mL to 100 ng/mL span is large; and the result was shown in Figure 5c, 5din our manuscript, the final LOD is determined to be 60 ng/mL according to Figure 5c, 5d.

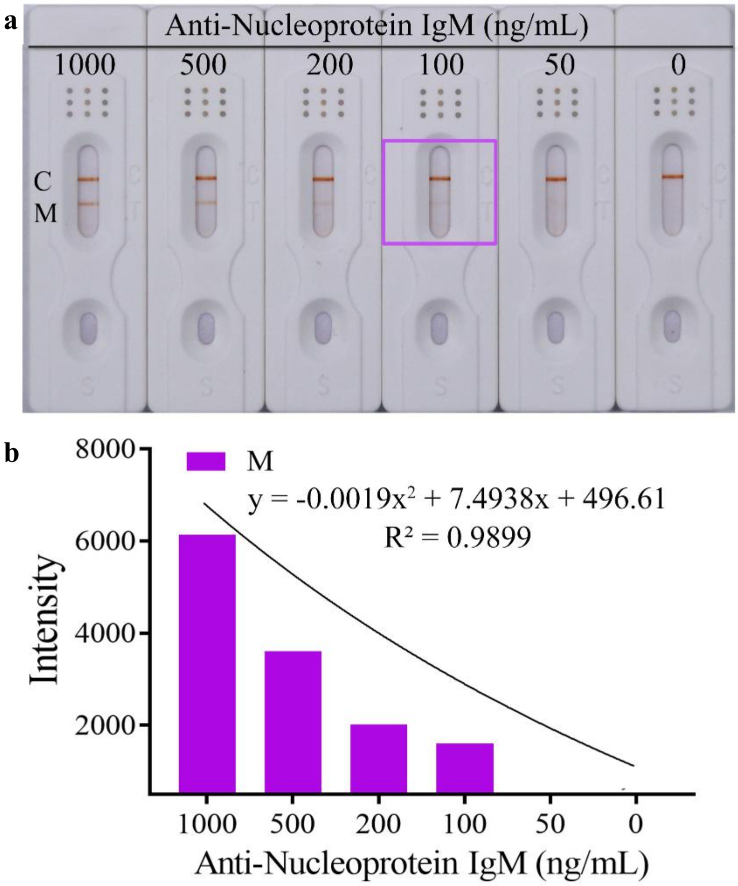

Figure S2 High concentration of anti-nucleoprotein IgM detection by the IgM kit. Result of detection for IgM (a) by naked eyes; Analysis result of detection for (b) by GraphPad Prism 8. C: Control line; M: Test line for IgM detection.

3 Basic information of clinical samples for figure 7 and Table 1

A total 72 clinical samples were detected: 19 positive sample of COVID-19 and 53 negative samples, and the negative samples contained 12 normal persons (Sample number 42-53) who had no cold symptoms within 2 weeks. The basic information of clinical samples are shown in table S1 - table S6, and the results are shown in figure S3 - figure S8.

The age and sex information of the positive samples are shown in table S1.

Table S1 Basic information of positive sample

**Number**	**Sex**	**Age**	**COVID-19 or not (+ / -)**	**Judgment method**	**Notes**
1	Female	70	+	RT-PCR	
2	Female	87	+	RT-PCR	
3	Male	58	+	RT-PCR	
4	Female	61	+	RT-PCR	
5	Male	90	+	RT-PCR	
6	Female	48	+	RT-PCR	
7	Male	43	+	RT-PCR	
8	Female	26	+	RT-PCR	
9	Female	88	+	RT-PCR	
11	Male	77	+	RT-PCR	
12	Male	57	+	RT-PCR	
13	Female	79	+	RT-PCR	
14	Female	78	+	RT-PCR	
15	Male	57	+	RT-PCR	
16	Female	46	+	RT-PCR	
17	Male	81	+	RT-PCR	False Negative
18	Female	79	+	RT-PCR	
19	Female	65	+	RT-PCR	
20	Male	57	+	RT-PCR	

The result of the positive samples for IgG detection are shown in figure S3, Naked eye results and auxiliary judgement are shown in figure S3a and figure S3b, respectively. The intensity of detection result for IgG are shown in table S2.

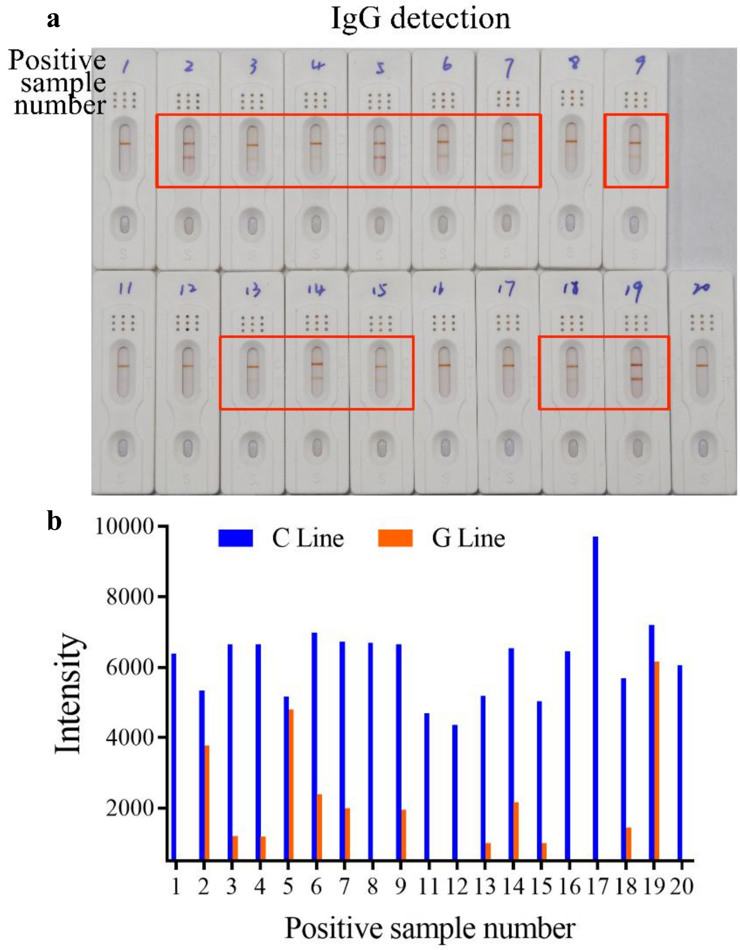

Figure S3 Positive sample detection results of anti-SARS-CoV-2 IgG (a) and auxiliary judgement (a). SARS-CoV-2, severe acute respiratory syndrome coronavirus 2, Cline: Control line; G line: Test line for IgG detection;

Table S2 Intensity results of positive samples for IgG detection

Sample number	1	2	3	4	5	6	7	8	9	
Color degree	—	+++	+	+	+++	++	++	—	+	
Sample number	11	12	13	14	15	16	17	18	19	20
Color degree	—	—	+	++	+	—	—	+	+++	—

The result of the positive samples for IgM detection are shown in figure S4, Naked eye results and auxiliary judgement are shown in figure S4a and figure S4b, respectively. The intensity of detection result for IgM are shown in table S3.

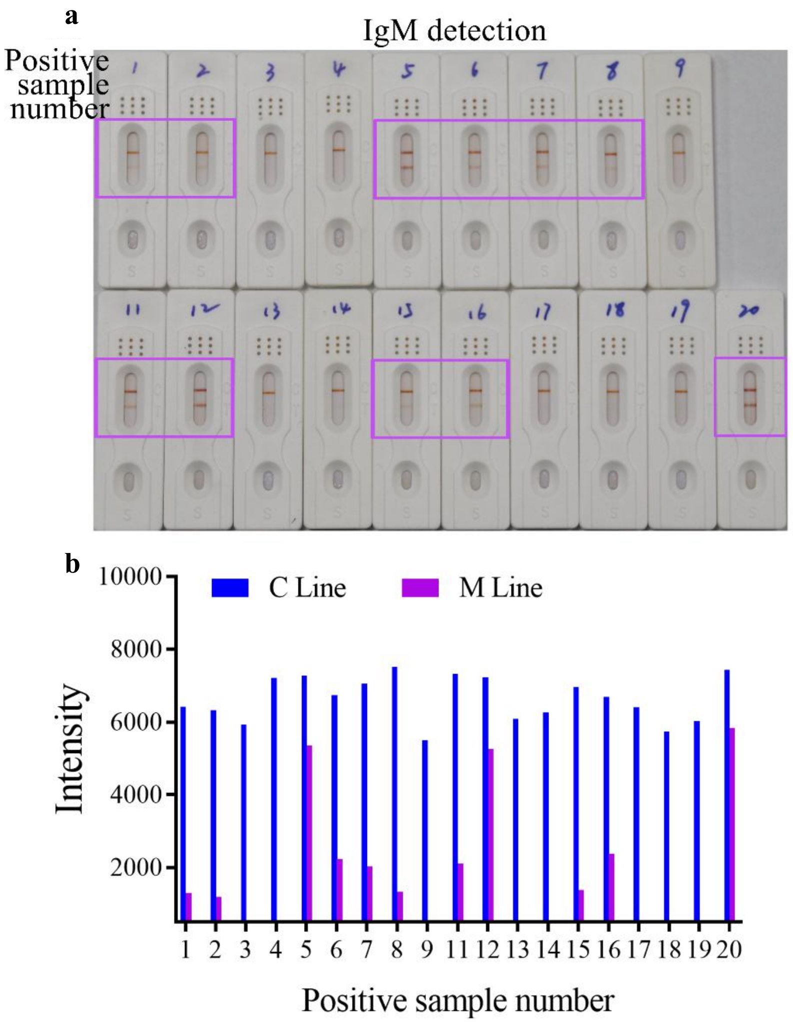

Figure S4 Positive sample detection results of anti-SARS-CoV-2 IgM (a) and auxiliary judgement (b). SARS-CoV-2, severe acute respiratory syndrome coronavirus 2, Cline: Control line; M line: Test line for IgM detection.

Table S3 Intensity results of positive samples for IgM detection

Sample number	1	2	3	4	5	6	7	8	9	
Color degree	+	+	−	−	+++	++	++	+	−	
Sample number	11	12	13	14	15	16	17	18	19	20
Color degree	++	+++	−	−	+	++	−	−	−	+++

The age and sex information of the negative samples are shown in table S4. Samples 42-53 are the serum of persons who have not had cold symptoms within two weeks.

Table S4 Negative sample information

**Number**	**Sex**	**Age**	**COVID-19 or not（+/-）**	**Judgment method**	**Notes**
1	Female	26	—	Clinical symptoms by doctor	
2	Female	38	—	Clinical symptoms by doctor	
3	Female	44	—	Clinical symptoms by doctor	
4	Female	44	—	Clinical symptoms by doctor	
5	Female	52	—	Clinical symptoms by doctor	
6	Female	53	—	Clinical symptoms by doctor	IgM false positive
7	Female	53	—	Clinical symptoms by doctor	
8	Female	84	—	Clinical symptoms by doctor	
9	Female	69	—	Clinical symptoms by doctor	
10	Female	69	—	Clinical symptoms by doctor	
11	Male	1	—	Clinical symptoms by doctor	
12	Male	31	—	Clinical symptoms by doctor	
13	Male	42	—	Clinical symptoms by doctor	
14	Male	49	—	Clinical symptoms by doctor	
15	Male	53	—	Clinical symptoms by doctor	
16	Male	53	—	Clinical symptoms by doctor	
17	Male	53	—	Clinical symptoms by doctor	
18	Male	51	—	Clinical symptoms by doctor	
19	Male	54	—	Clinical symptoms by doctor	
20	Male	55	—	Clinical symptoms by doctor	

Number	Sex	Age	COVID-19 or not（ + / - ）	Judgment method	Notes
21	Male	56	—	Clinical symptoms by doctor	IgG false positive
22	Female	31	—	Clinical symptoms by doctor	
23	Female	40	—	Clinical symptoms by doctor	
24	Female	43	—	Clinical symptoms by doctor	
25	Female	48	—	Clinical symptoms by doctor	
26	Female	59	—	Clinical symptoms by doctor	
27	Female	60	—	Clinical symptoms by doctor	
28	Female	16	—	Clinical symptoms by doctor	
29	Male	16	—	Clinical symptoms by doctor	
30	Male	18	—	Clinical symptoms by doctor	
31	Male	31	—	Clinical symptoms by doctor	
32	Male	32	—	Clinical symptoms by doctor	
33	Male	46	—	Clinical symptoms by doctor	
34	Male	46	—	Clinical symptoms by doctor	
35	Male	49	—	Clinical symptoms by doctor	
37	Male	50	—	Clinical symptoms by doctor	
38	Male	52	—	Clinical symptoms by doctor	
39	Male	60	—	Clinical symptoms by doctor	
40	Male	57	—	Clinical symptoms by doctor	
41	Male	57	—	Clinical symptoms by doctor	

Number	Sex	Age	COVID-19 or not（+/-）	Judgment method	Notes
42	Male	25	—	Clinical symptoms by doctor	
43	Female	25	—	Clinical symptoms by doctor	
44	Male	26	—	Clinical symptoms by doctor	
45	Female	25	—	Clinical symptoms by doctor	
46	Female	26	—	Clinical symptoms by doctor	
47	Male	26	—	Clinical symptoms by doctor	
48	Male	23	—	Clinical symptoms by doctor	
49	Female	21	—	Clinical symptoms by doctor	
50	Male	25	—	Clinical symptoms by doctor	
51	Female	23	—	Clinical symptoms by doctor	
52	Female	23	—	Clinical symptoms by doctor	
53	Male	31	—	Clinical symptoms by doctor	

The result of the negative samples for IgG detection are shown in figure S5 and figure S6. Naked eye results are shown in figure S5, auxiliary judgement are shown in figure S6. The intensity of detection result for IgG are shown in table S5.

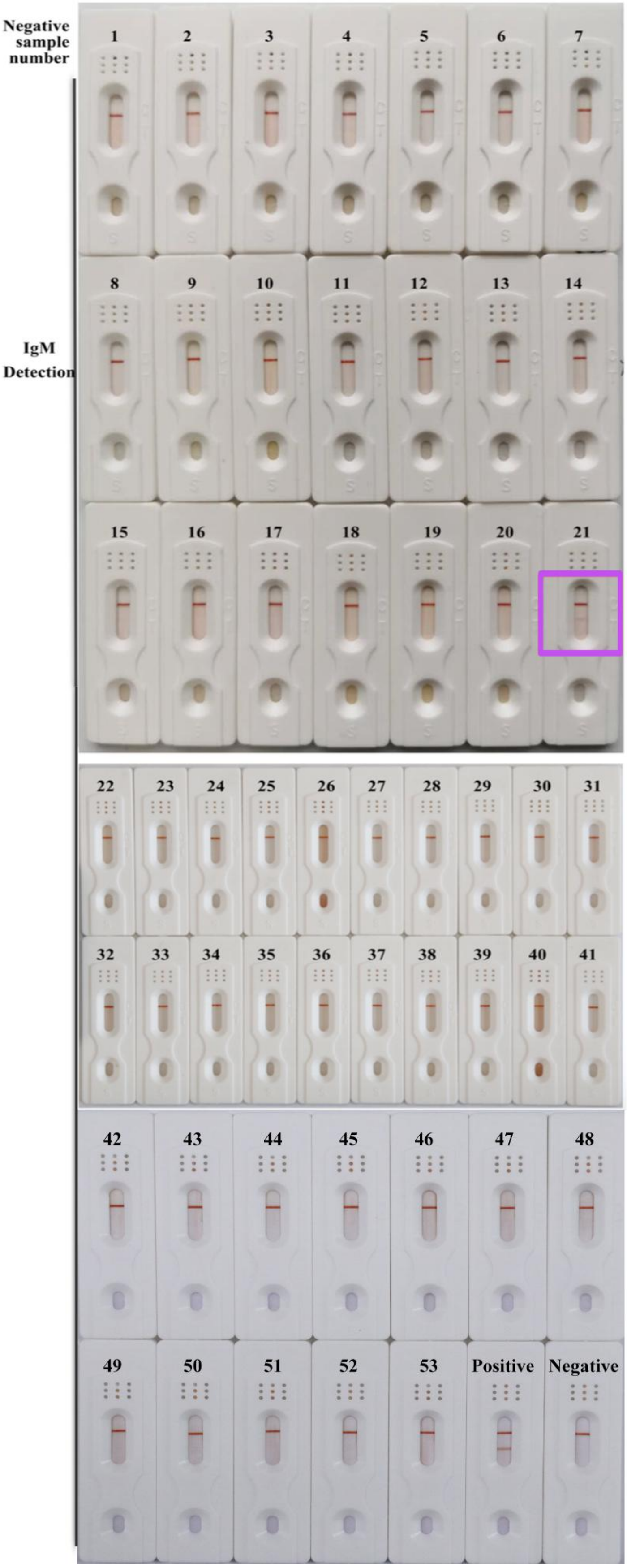

Figure S5 **IgG** detection results of all negative samples

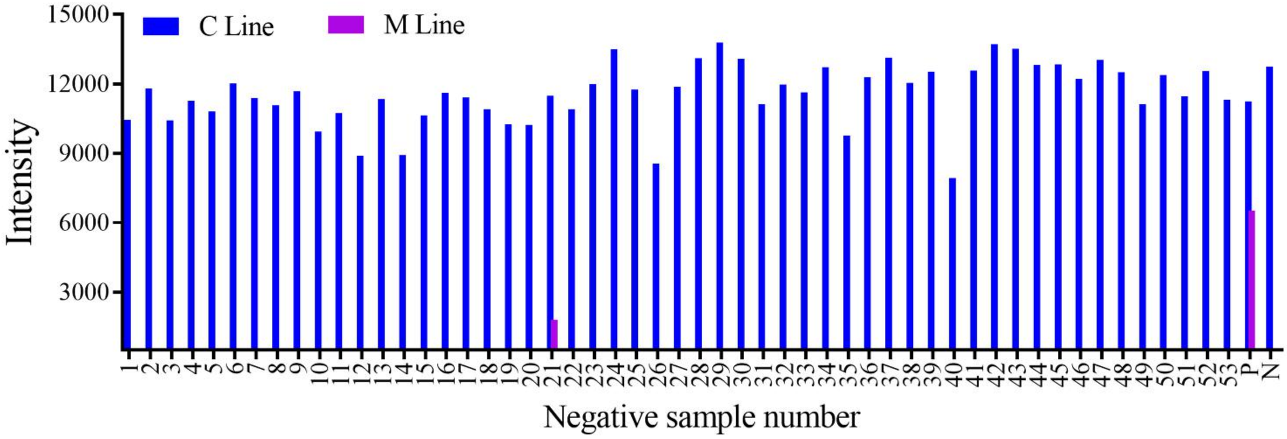

Figure S6 Auxiliary judgement of negative sample detection results of anti-SARS-CoV-2 IgG. SARS-CoV-2, severe acute respiratory syndrome coronavirus 2, C line: Control line; G line: Test line for IgG detection; P: Positive; N: Negative.

Table S5 Intensity results of negative samples for IgG detection

Sample number	1	2	3	4	5	6	7	8	9	10
Color degree	—	—	—	—	—	++	—	—	—	—
Sample number	11	12	13	14	15	16	17	18	19	20
Color degree	—	—	—	—	—	—	—	—	—	—
Sample number	21	22	23	24	25	26	27	28	29	30
Color degree	—	—	—	—	—	—	—	—	—	—
Sample number	31	32	33	34	35	36	37	38	39	40
Color degree	—	—	—	—	—	—	—	—	—	—
Sample number	41	42	43	44	45	46	47	48	49	50
Color degree	—	—	—	—	—	—	—	—	—	—
Sample number	51	52	53	Positive	Negative					
Color degree	—	—	—	+++	—					

The result of the negative samples for IgG detection are shown in figure S7 and figure S8. Naked eye results are shown in figure S7, auxiliary judgement are shown in figure S8. The intensity of detection result for IgG are shown in table S6.

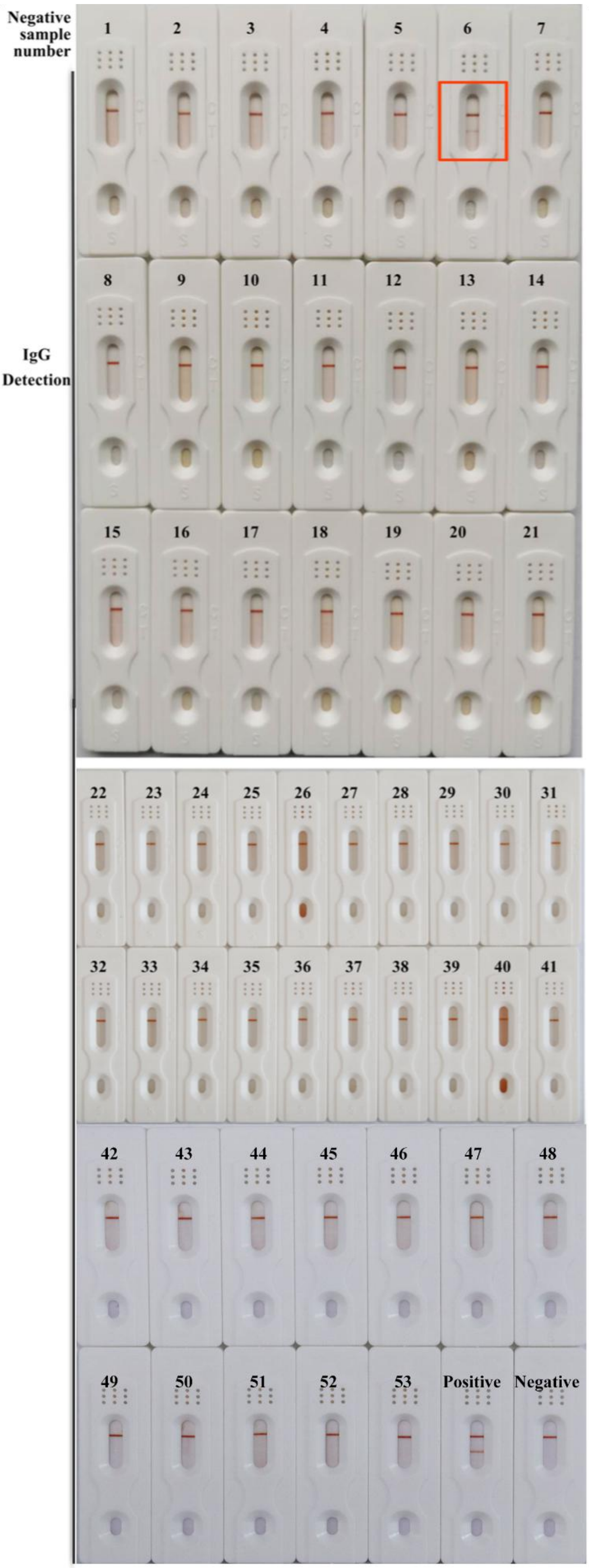

Figure S7 **IgM** detection results of all negative samples

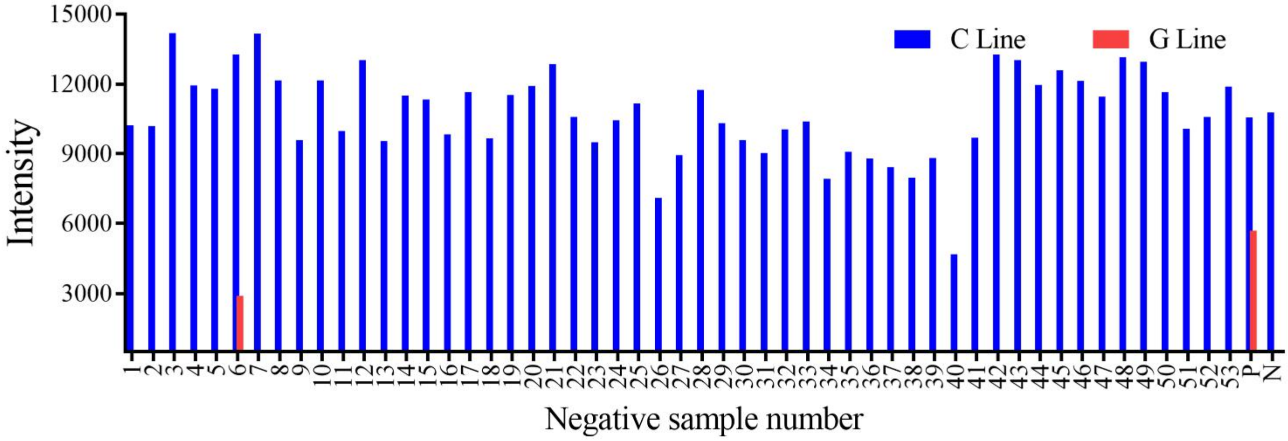

Figure S8 Auxiliary judgement of negative sample detection results of anti-SARS-CoV-2 IgM. SARS-CoV-2, severe acute respiratory syndrome coronavirus 2, C line: Control line; M line: Test line for IgM detection; P: Positive; N: Negative.

Table S6 Intensity results of negative samples for IgM detection

Sample number	1	2	3	4	5	6	7	8	9	10
Color degree	—	—	—	—	—	—	—	—	—	—
Sample number	11	12	13	14	15	16	17	18	19	20
Color degree	—	—	—	—	—	—	—	—	—	—
Sample number	21	22	23	24	25	26	27	28	29	30
Color degree	+	—	—	—	—	—	—	—	—	—
Sample number	31	32	33	34	35	36	37	38	39	40
Color degree	—	—	—	—	—	—	—	—	—	—
Sample number	41	42	43	44	45	46	47	48	49	50
Color degree	—	—	—	—	—	—	—	—	—	—
Sample number	51	52	53	Positive	Negative					
Color degree	—	—	—	+++	—					

4 Detection result for serum after the second vaccine injection by this kit

34 serum samples after second vaccination with attenuated vaccine were detected, 31 samples showed positive for IgG (Figure S9a) and/or IgM (Figure S9b) , except sample 8, 11 and 22. The response rate to the vaccine was 91.18%.

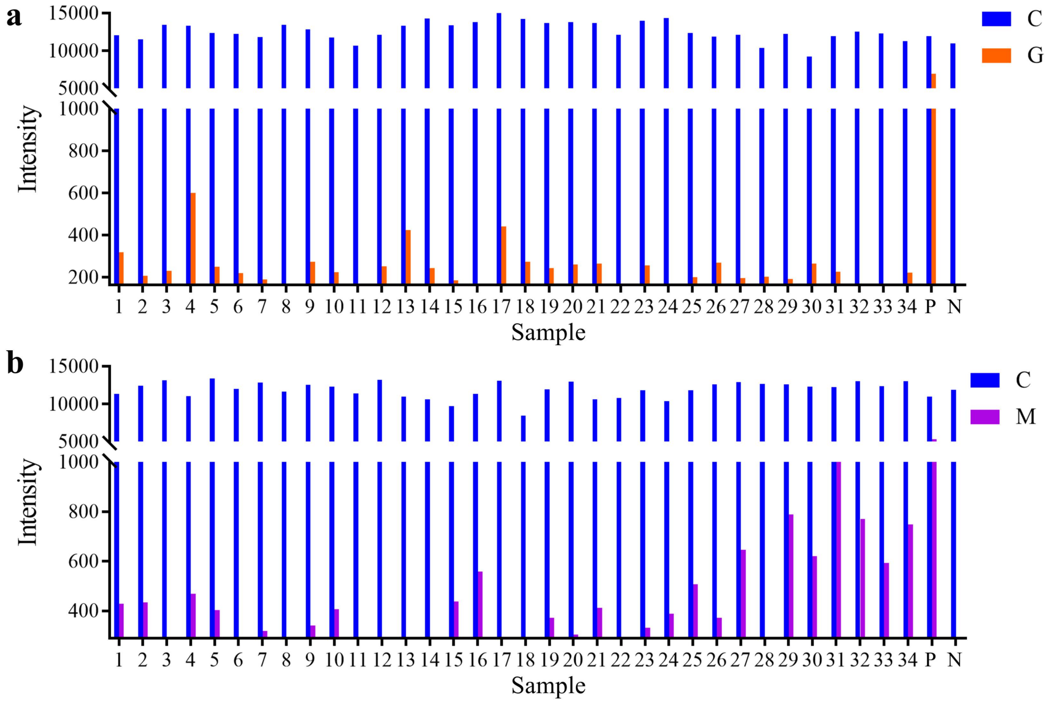

Figure S9 Detection result for serum after the second vaccination. (a) Detection result for IgG, (b)Detection result for IgM. P: positive, N:negative.
